# Supplementary material for: Crossmodal sensory neurons based on high-performance flexible memristors for human-machine in-sensor computing system
Source: Nat Commun. 2024 Aug 23;15:7275. doi: 10.1038/s41467-024-51609-x (PMC11344147; doi:10.1038/s41467-024-51609-x)
Supplement: Supplementary file 1 — Supplementary information [file 41467_2024_51609_MOESM1_ESM.pdf]

Supplementary Information

Crossmodal sensory neurons based on high-performance flexible memristors for human-machine in-sensor computing system

Zhiyuan Li[1,2†], Zhongshao Li[3,4†], Wei Tang[1], Jiaping Yao[1], Zhipeng Dou[5], Junjie Gong[1], Yongfei Li[1], Beining Zhang[1], Yunxiao Dong[1], Jian Xia[1], Lin Sun[5], Peng Jiang[5], Xun Cao[3,4*], Rui Yang[1,2*], Xiangshui Miao[1,2*], and Ronggui Yang[6]

[1]School of Integrated Circuits, Huazhong University of Science and Technology, Wuhan 430074, China.

[2]Hubei Yangtze Memory Laboratories, Wuhan 430205, China.

[3]State Key Laboratory of High Performance Ceramics and Superfine Microstructure, Shanghai Institute of Ceramics, Chinese Academy of Sciences, Shanghai, China.

[4]Center of Materials Science and Optoelectronics Engineering, University of Chinese Academy of Sciences, Beijing, China.

[5]State Key Laboratory of Catalysis, CAS Center for Excellence in Nanoscience, Dalian Institute of Chemical Physics, Chinese Academy of Sciences, Dalian 116023, China.

[6]State Key Laboratory of Coal Combustion, School of Energy and Power Engineering, Huazhong University of Science and Technology, Wuhan 430074, China.

[†]These authors contributed equally.

[*]E-mail: yangrui@hust.edu.cn; cxun@mail.sic.ac.cn; miaoxs@hust.edu.cn

This **Supplementary Information** includes:

Supplementary Figures 1-28;

Supplementary Tables 1-4;

Supplementary Notes 1-5.

Supplementary Figures

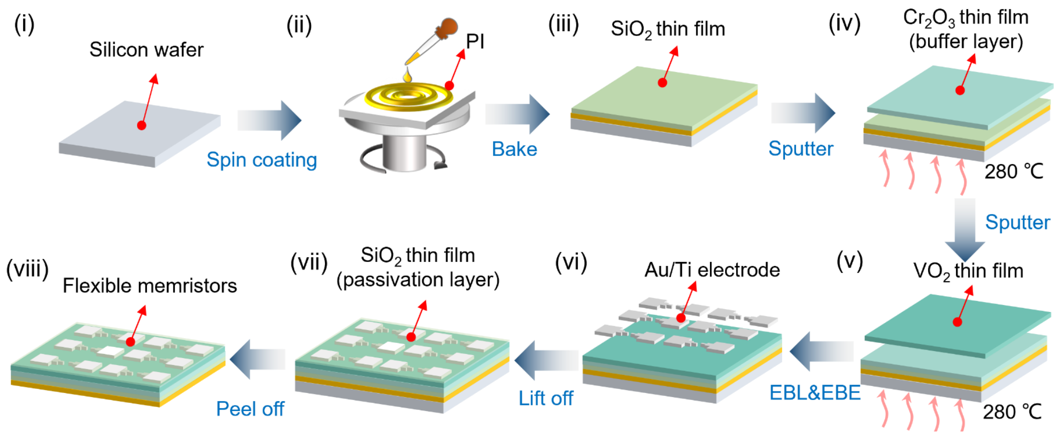

Supplementary Figure 1. The detailed fabrication process flow for flexible VO_2 memristors.

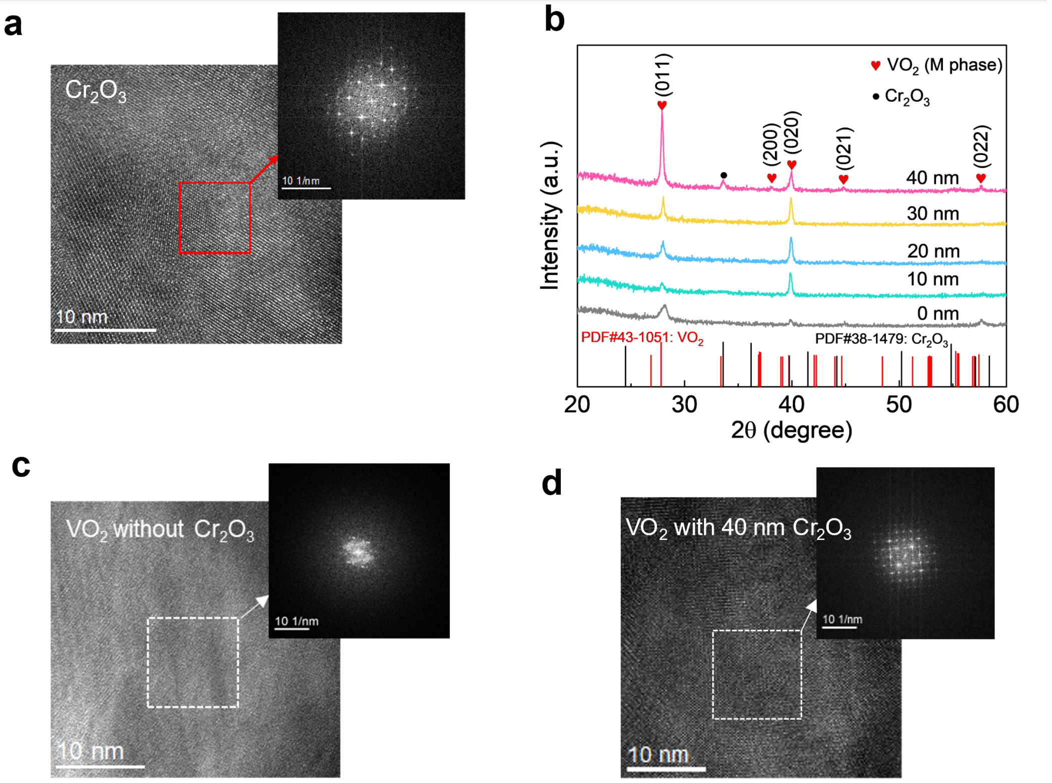

Supplementary Figure 2. Characterization of the Cr_2O_3 film and VO_2 film. (a) Cross-sectional transmission electron microscopy (TEM) and electron diffraction pattern image of the Cr_2O_3 film. **(b)** X-ray diffraction (XRD) spectra of the VO_2 thin film grown on the Cr_2O_3 buffer layers. TEM images and electron diffraction patterns of the VO_2 film without **(c)** /with **(d)** Cr_2O_3 layer.

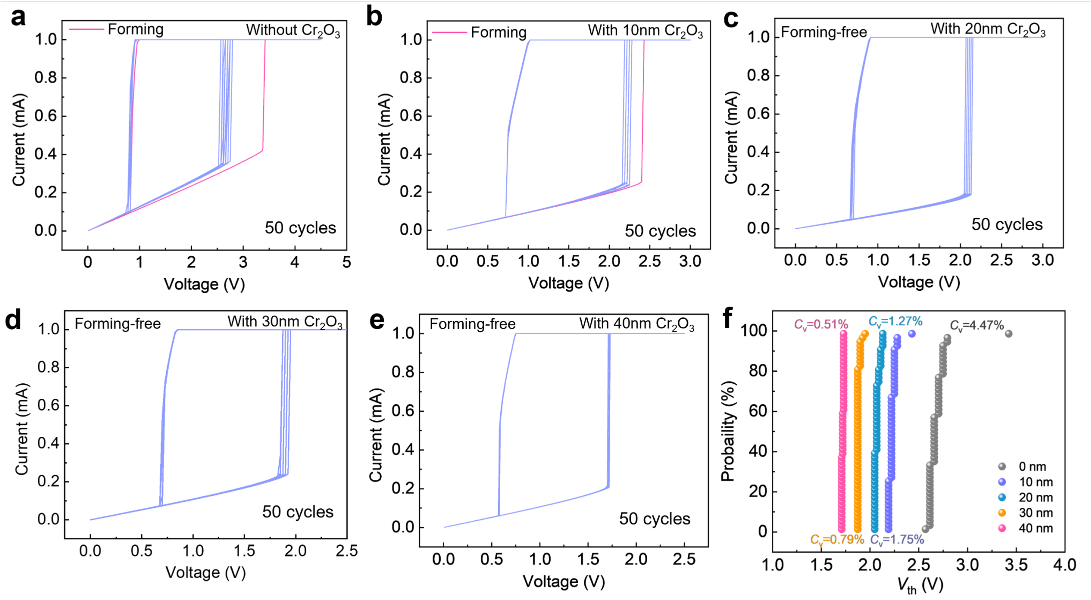

Supplementary Figure 3. *I-V* characteristics of the VO_2 devices grown with Cr_2O_3 buffer layer of (a) 0 nm, (b) 10 nm, (c) 20 nm, (d) 30 nm, and (e) 40 nm. (f) The cycle-to-cycle (C2C) threshold voltage (V_{th}) distribution of VO_2 device with different thickness of Cr_2O_3 buffer layer. For different devices, 50 repeated switching cycles were measured. Without the buffer layer, the crystallinity of the deposited VO_2 films is insufficient, necessitating a forming process to create a VO_2 phase transition channel. This formed channel can be random and lead to device instability. The high crystalline quality of VO_2 also leads to markedly improved C2C uniformity. With the increase in buffer layer thickness, the C2C variation in V_{th} decreases from 4.47% to 0.51%. The VO_2 memristor based on 40-nm Cr_2O_3 demonstrates extremely low C2C variability due to its high crystalline structure.

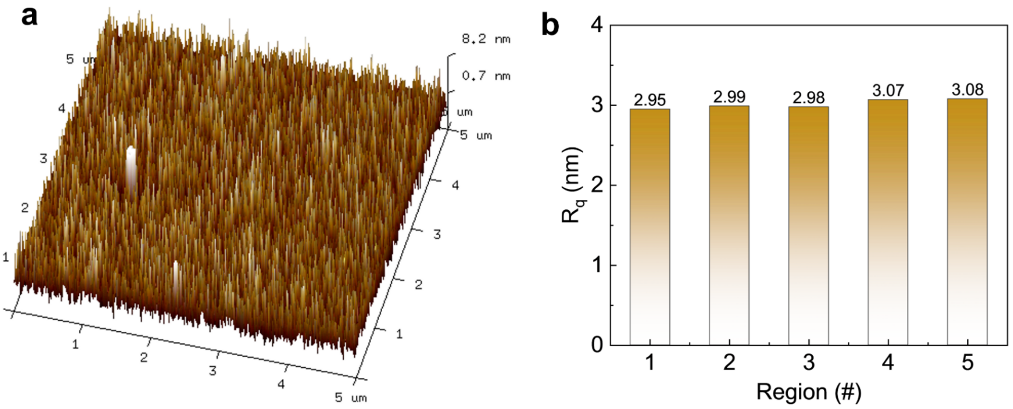

Supplementary Figure 4. Surface morphology of the VO_2 film. **(a)** Atomic force microscope (AFM) electron micrographs of VO_2 film. **(b)** root mean square roughness (R_q) of five randomly selected regions on the film.

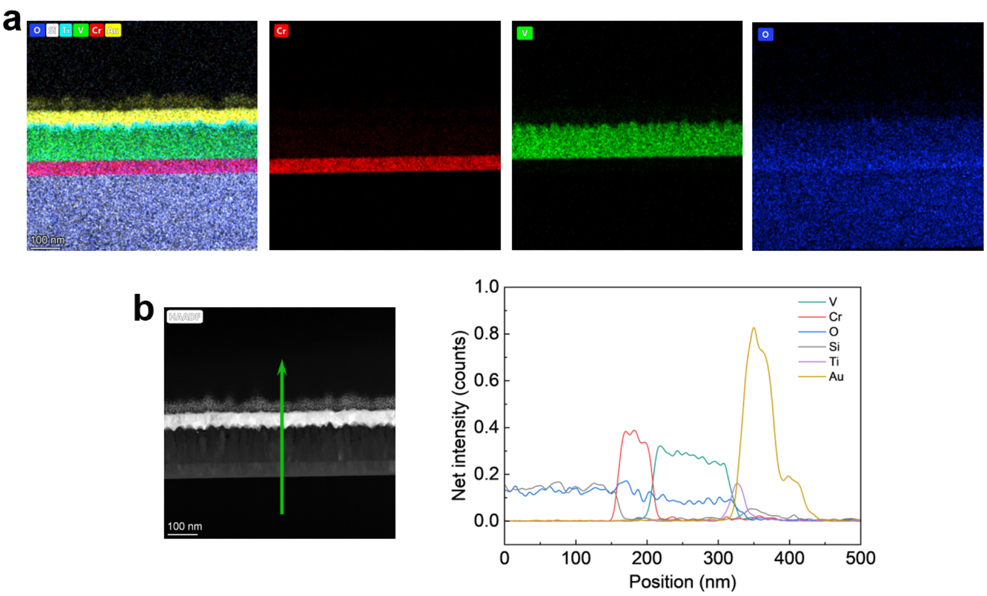

Supplementary Figure 5. Characterization of the VO_2 memristor. (a) Cross-sectional energy dispersive X-ray spectroscopy (EDS) mapping of Cr, V, and O elements in the device. **(b)** EDS elemental line profile of the device.

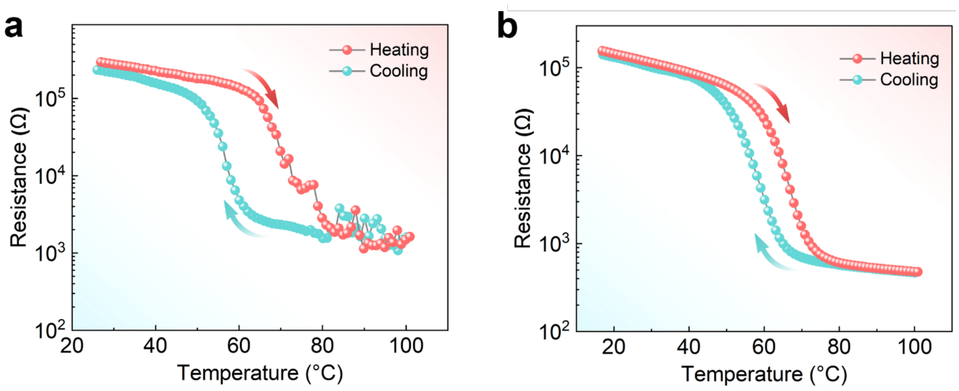

Supplementary Figure 6. Temperature-dependent resistance of the VO_2 films deposited without (a)/with (b) Cr_2O_3 buffer. The as-grown VO_2 film without Cr_2O_3 buffer layer shows an unstable phase transition process. After introducing the Cr_2O_3 buffer layer, the temperature-dependent resistance curve of the VO_2 film exhibits a stable change in ~3 orders of magnitude. The noise in the resistance signal at high temperatures (80 to 100 °C) is likely due to electrical noise or device issues, rather than the phase transition process. This signal is not typical in high-quality VO_2 devices. For VO_2 films deposited without a buffer layer, the increased noise is due to the mixture of VO_2(M) and VO_2(B) phases from direct deposition at 280°C, leading to inhomogeneous phase transition behavior, and due to the low crystalline quality, increased surface roughness, and poor electrode contact at high temperatures.

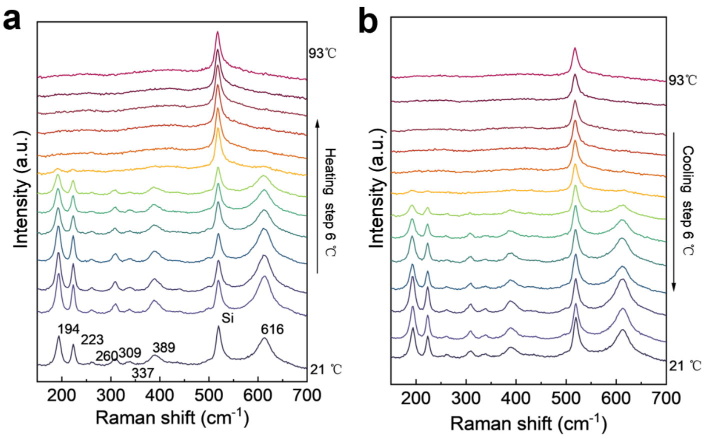

Supplementary Figure 7. Structural phase transition of VO_2 during heating and cooling processes. *In-situ* Raman spectrum of the VO_2 film during heating **(a)** and cooling **(b)**. A series of peaks at 194, 223, 260, 309, 337, 389, 499, and 616 cm^{-1} are corresponding to the characteristic vibration modes of M phase VO_2[1, 2]. No impurity peak is observed, indicating pure VO_2 (M) films have been fabricated with the Cr_2O_3 buffer layer at low temperature. The characteristic Raman peaks of VO_2 (M) decrease gradually with temperature increasing and disappear completely at 65 °C. As the temperature decreases, the disappearing peaks rise gradually, proving the appearance of the M phase. These results indicate a structural phase transition from low-temperature M phase to high-temperature R phase[3], which is consistent with the temperature-dependent resistance results. All samples were cooled back to room temperature after heating measurement.

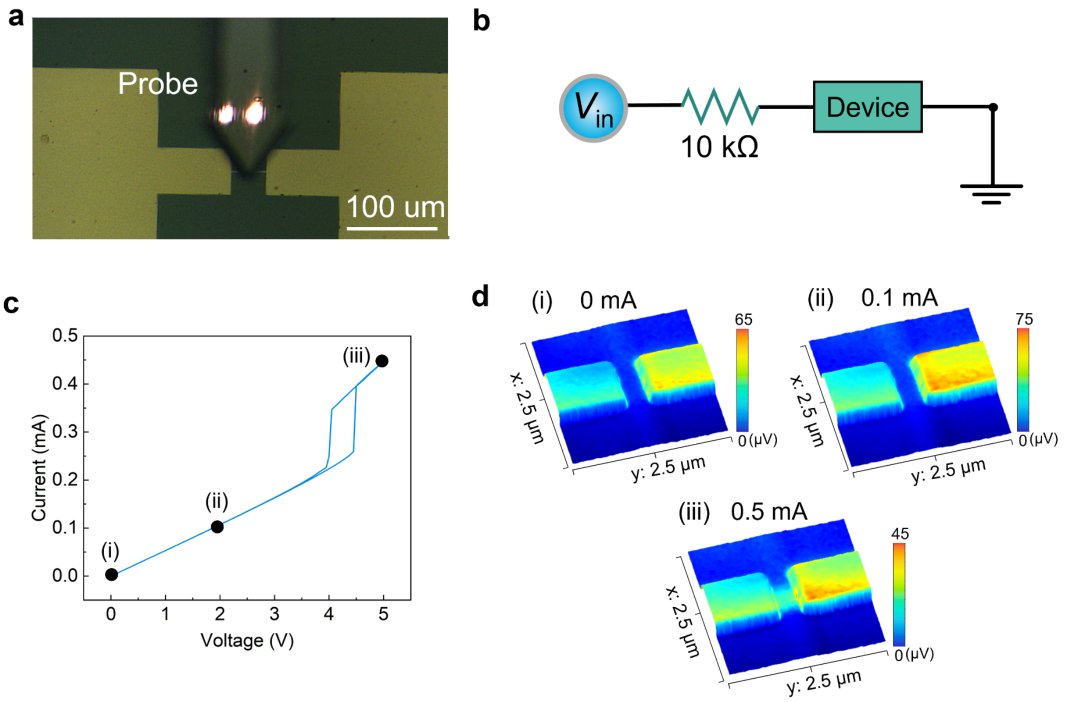

Supplementary Figure 8. *In-situ* scattering-type scanning near-field optical microscopy (s-SNOM) mapping measurement in VO_2 memristor. (a) Photograph of s-SNOM measurement. **(b)** Electrical test circuit diagram of VO_2 memristor during in-situ s-SNOM testing process. **(c)** *I-V* characteristics of VO_2 memristor with a 10 kΩ series resistor. **(d)** *In-situ* 3D optical amplitude map of the device area at different current values for the same device. The states of i and ii correspond to the high resistance state (HRS) and low resistance state (LRS), respectively. When the applied voltage (state ii) is smaller than the V_{th}, the device remains in a HRS with minimal SNOM signal variation despite temperature changes in the channel area. Once the applied voltage exceeds the V_{th}, the device transitions to the LRS, and significant SNOM signal changes are observed. These changes are primarily driven by the phase transition in the VO_2 film, which alters its optical properties.

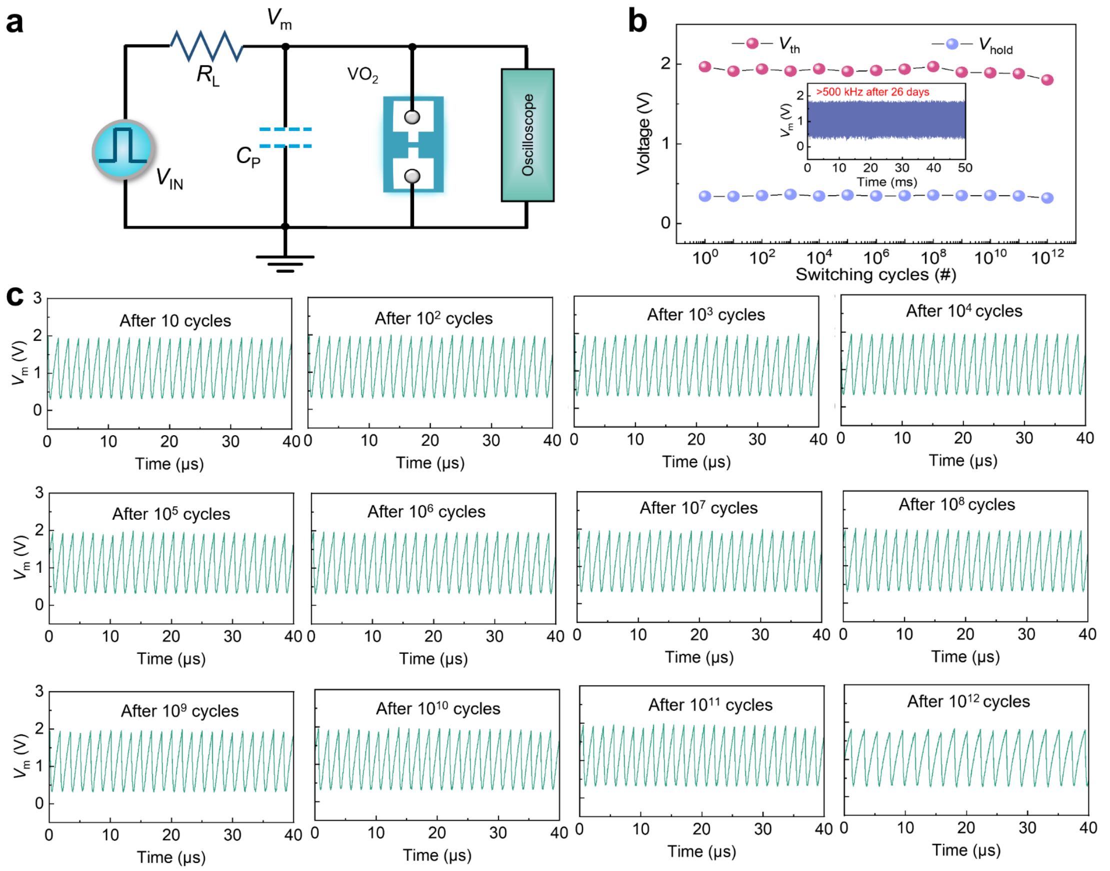

Supplementary Figure 9. Endurance circuit measurement of the VO_2 memristor. **(a)** Illustration of proposed endurance test method with a memristor-based oscillator circuit and an oscilloscope (counter unit). V_{in} is set as 5 V, and R_L is set as 5 kΩ. **(b)** V_{th} and V_{hold} extracted from the oscillating behavior of the circuit. The memristor still work well even after 26 days of continuous oscillation. The standard deviations of V_{th} and V_{hold} are 0.0432 V and 0.0106V, respectively. The coefficient of variation ($C_v = \sigma/\mu$, σ is the standard deviation and μ is the mean value) in V_{th} and V_{hold} are as low as 2.26% and 3.05%, respectively. **(c)** Stable oscillating behavior of the VO_2 memristor circuit during 10^{12} switching cycles. The number of switching was calculated by the following equation: $N = f_{fri} \times T$, where f_{fri}, the average oscillation frequency, is greater than 500 kHz, and T is total oscillation time of 2.25×10^6 seconds, resulting in 1.1×10^{12} cycles.

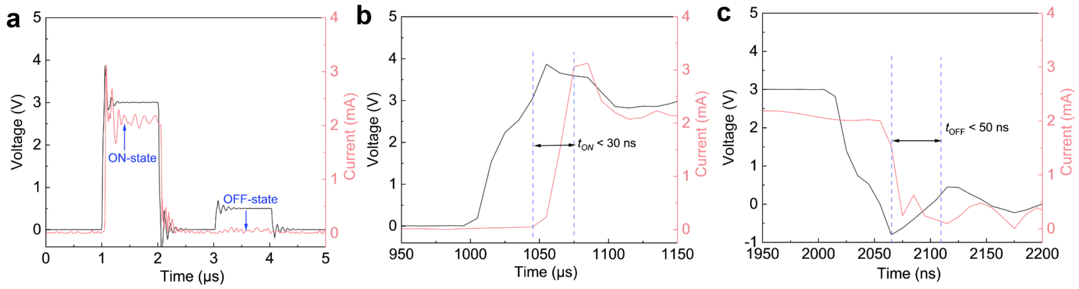

Supplementary Figure 10. Transient characterization the VO_2 memristors. (a) Dynamical transient response of the device under two consecutive voltage pulses (3 V, 1 us and 0.5 V, 1 us). Enlarged view of ON **(b)** and OFF **(c)** switching curves. The device shifts from OFF state to ON state within 30 ns and returns to OFF state within 50 ns.

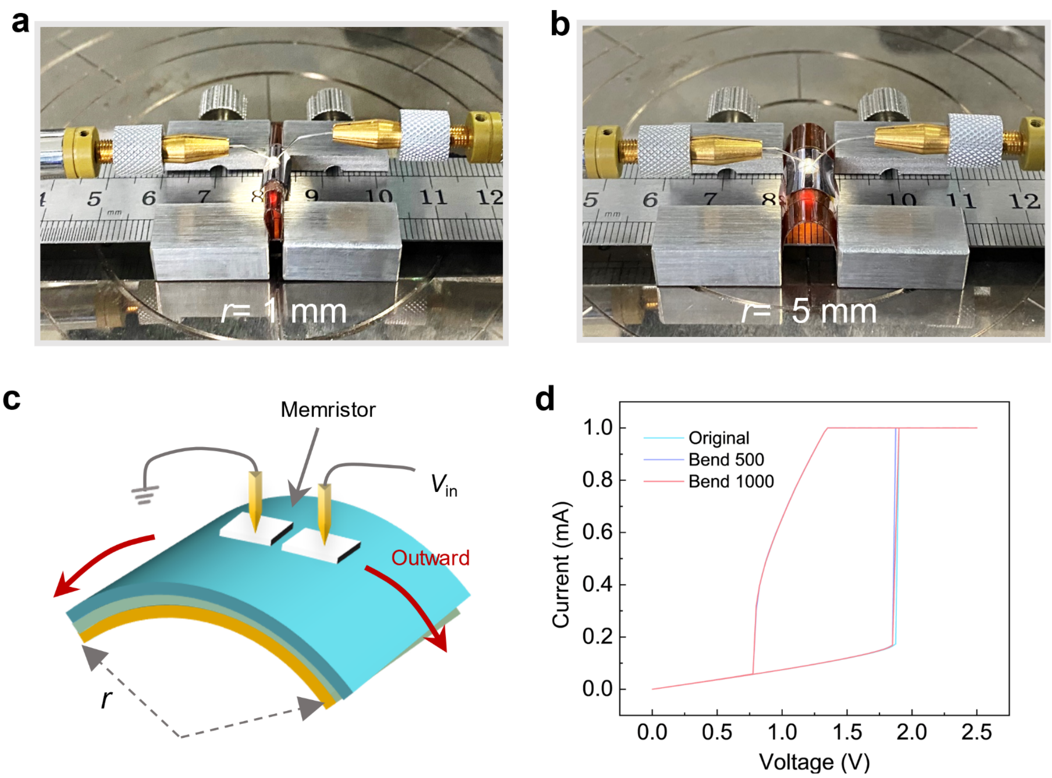

Supplementary Figure 11. Flexibility characterization of the flexible VO_2 memristor. Photograph of the flexible device on sliding fixtures at a bending radius of 1 mm **(a)** and 5 mm **(b)**. **(c)** A schematic diagram of the flexible device undergoing bending. The bending orientation is an out-of-plane bending (red arrows), meaning the device is flexing along a curvature. **(d)** *I*–*V* characteristics of the flexible VO_2 device under consecutive 1000 mechanical bending cycles show no noticeable deterioration. The bending radius is 5 mm.

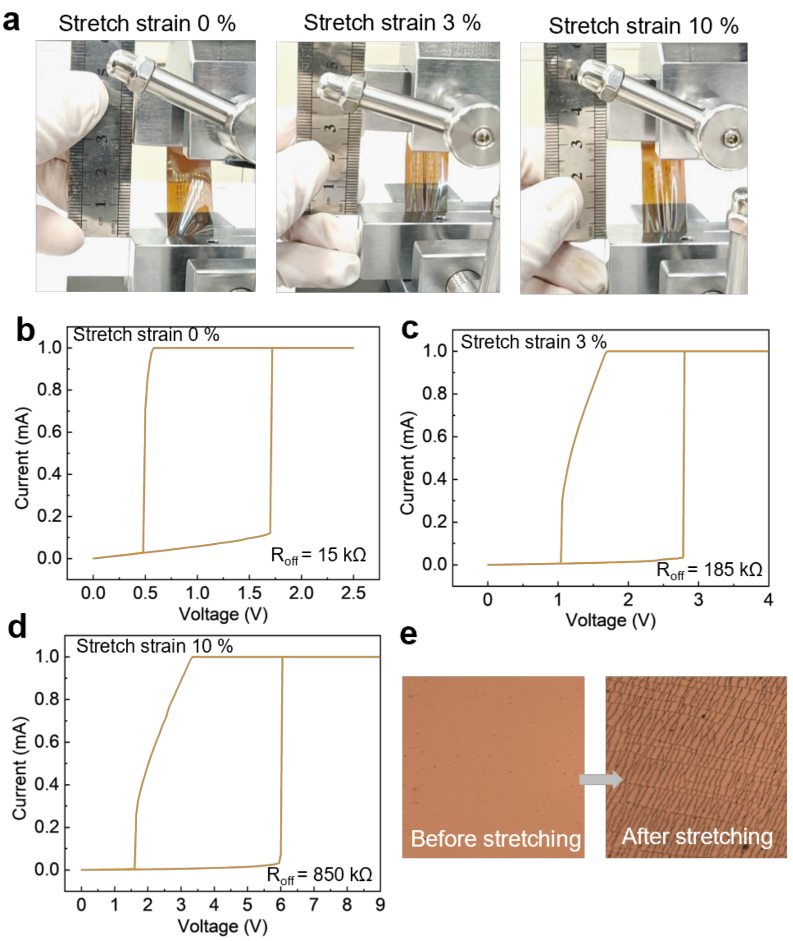

Supplementary Figure 12. Characterization of the flexible VO_2 memristor under stretching conditions. (a) Optical images showing flexible VO_2 memristors under strains of 0% (initial), 3%, and 10% @ y direction. **(b-d)** *I-V* curves of the flexible VO_2 memristor at strains of 0-10%. **(e)** Surface morphology of the VO_2 film before and after stretching (10%). The flexible VO_2 memristors maintain switching behavior under tensile strains up to 10%. The V_{th} and initial resistance (R_{off}) of the flexible device increase with tensile strain due to permanent deformation and rupture of the VO_2 thin film and electrodes on the PI substrate. In practical wearable application scenarios, he stretching strain range of 0-10% and strong resistance to stretching that our PI-based VO_2 memristors can accommodate is sufficient for many flexible device applications[4,5].

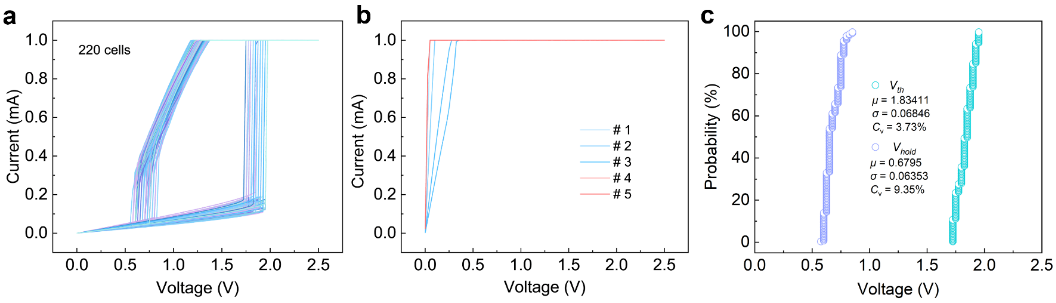

Supplementary Figure 13. Device-to-device (D2D) variation of the flexible VO_2 memristors. (**a, b**) *I-V* curves of 225 different flexible VO_2 devices. Here we note that 5 of the total 255 measured devices do not exhibit switching behavior. (**c**) Cumulative probability of V_{th} and V_{hold} in 220 flexible devices. The average V_{th} and V_{hold} are 1.83 V and 0.68 V, respectively. The standard deviations of V_{th} and V_{hold} are 0.068 V and 0.064 V, respectively. The D2D variation of the V_{th} and V_{hold} are 3.73% and 9.35%, respectively.

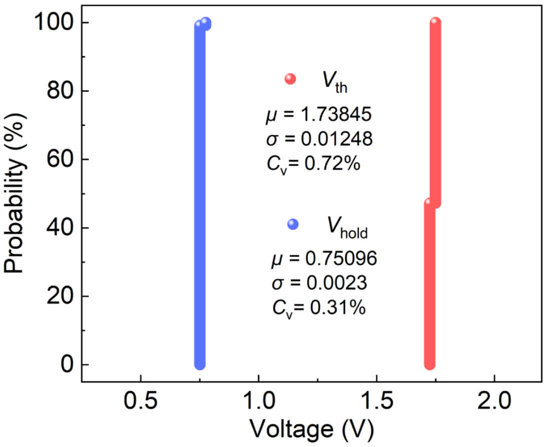

Supplementary Figure 14. C2C variation of the VO_2 memristor. Cumulative probability of V_{th} and V_{hold} in 2000 repeated DC sweeping. The average V_{th} and V_{hold} are 1.74 V and 0.75 V, respectively. The standard deviations of V_{th} and V_{hold} are 0.012 V and 0.002 V, respectively. The C2C variation in V_{th} and V_{hold} are as low as 0.72% and 0.31%, respectively.

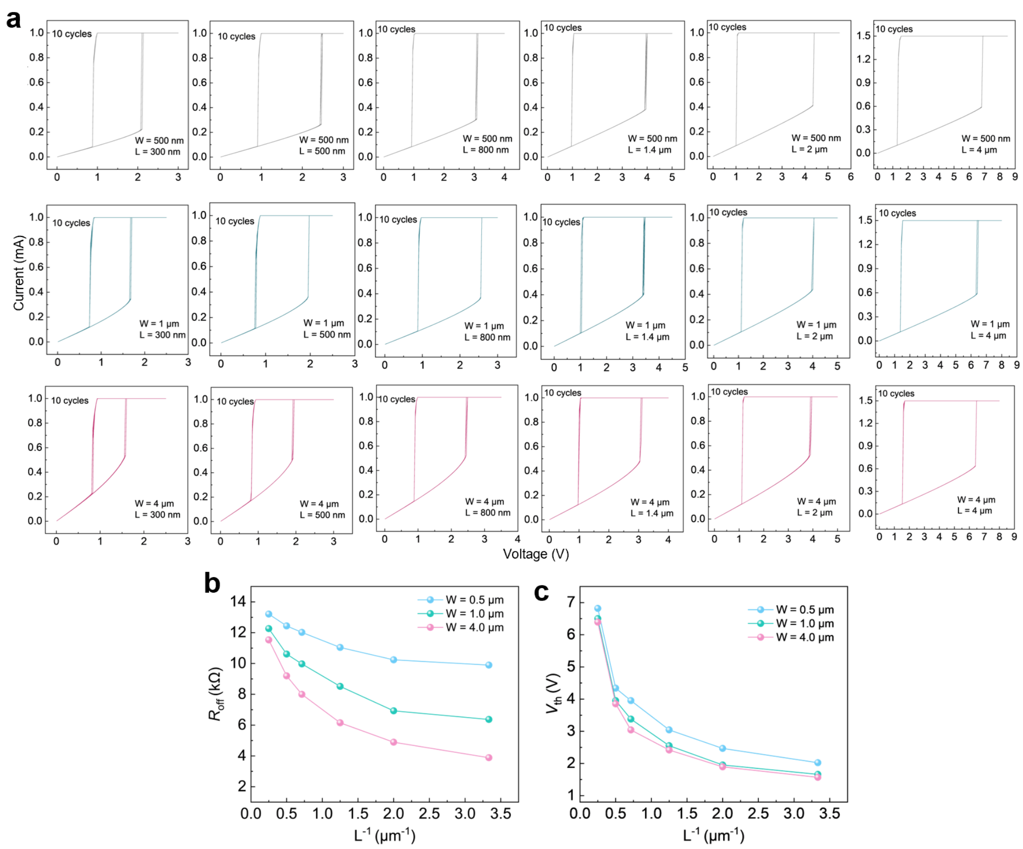

Supplementary Figure 15. Threshold switching behavior of the flexible planar VO_2 device under different active area size. (a) The *I-V* curves for different device size, with channel lengths from 300 nm to 4 μm and widths from 500 nm to 4 μm. Each size was tested for 10 cycles, exhibiting stable threshold switching behaviors. R_{off} **(b)** and V_{th} **(c)** for device with varying channel lengths and widths. The change in device area affects the total area over which the IMT must propagate and affects the TS performance. The R_{off} increases with increasing channel lengths and decreases with increasing channel widths. V_{th} will increase along with higher R_{off}, as more energy is required to achieve the same level of joule heating necessary to induce the IMT. Consequently, a higher V_{th} is observed. These results demonstrate a design space and pathway toward realizing low-power operation and high-density integration.

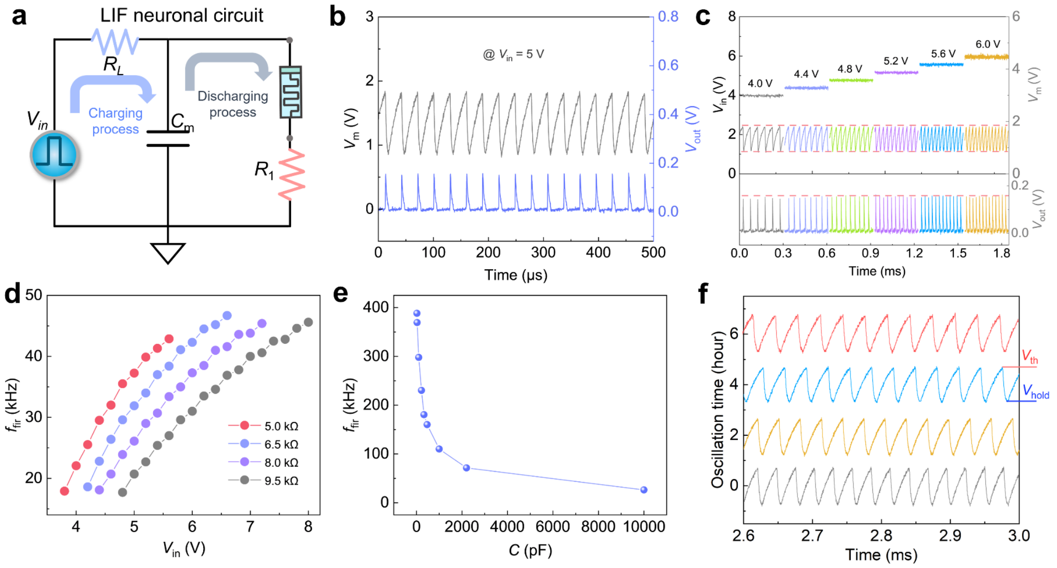

Supplementary Figure 16. The artificial leaky integrate and fire (LIF) spiking neuron based on VO_2 memristor. (a) Schematic circuit diagram of LIF neuron based on VO_2 memristor. **(b)** The artificial neuron response under an input voltage (V_{in}) of 5 V/800 μs, R_L = 5 kΩ, R_1 = 50 Ω, and C_m = 10 nF. The charging and discharging processes can be clearly observed. **(c)** Response of the artificial neuron with increasing V_{in}. **(d)** Statistics of the output firing frequency (f_{fri}) and V_{in} relationship under different R_L, showing a positive relationship. **(e)** Statistics relationship of the f_{fri} and C_m. A longer C_m constant time decreases the f_{fri}. **(f)** Stable neuronal oscillation characteristics during 6 hours test. Additional discussions are provided in Supplementary Note 2.

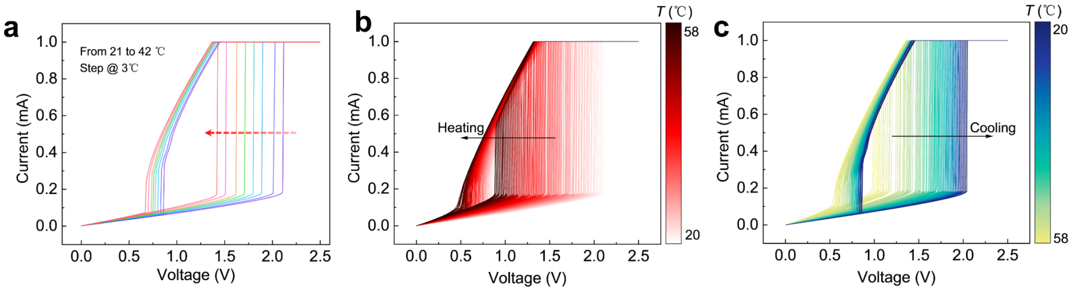

Supplementary Figure 17. Temperature-dependent *I-V* switching characteristics of the VO_2 memristor under heating (a, b) and cooling (c). With increasing temperature, the window of V_{th} -V_{hold} shrinks gradually. With decreasing temperature, the window of V_{th} -V_{hold} expands gradually.

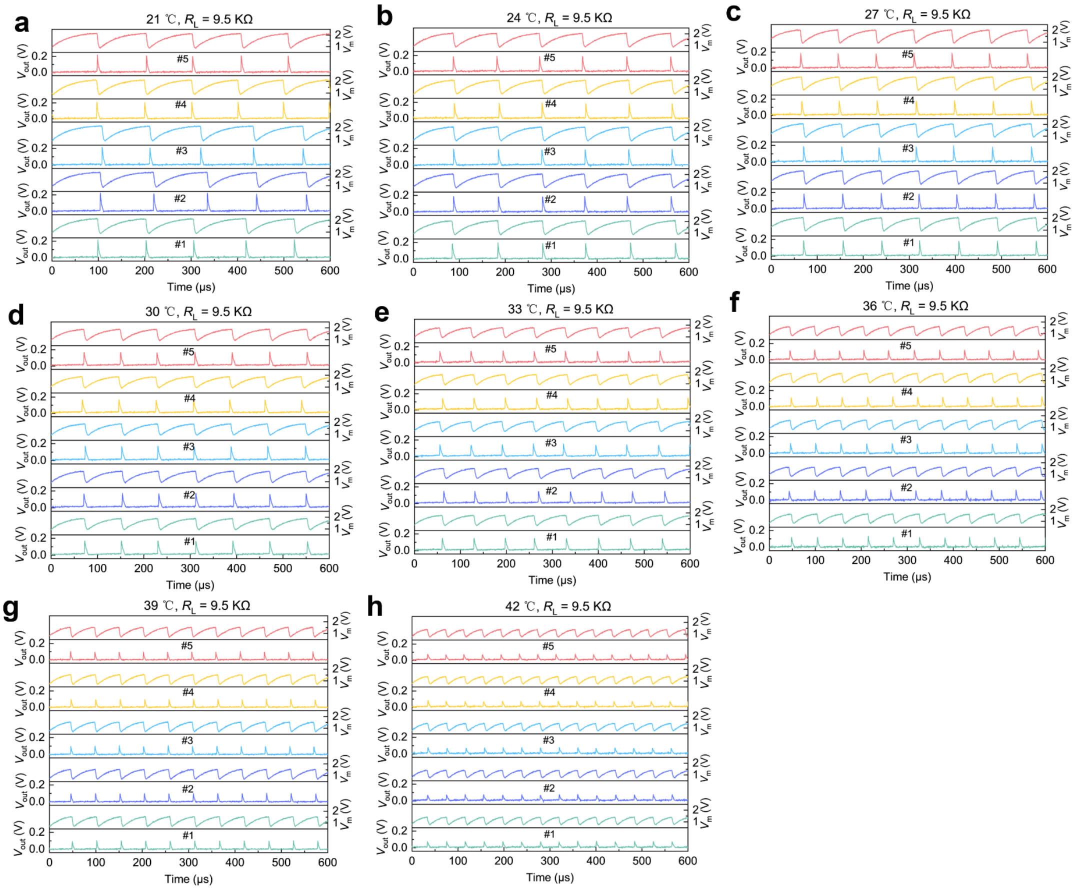

Supplementary Figure 18. The output responses of the spiking temperature sensory neuron at different temperatures with series resistance R_L=9.5 kΩ. (a-h) Shown here are the responses of V_m and V_{out} to different temperature (21-42 ℃). Test five times under each condition.

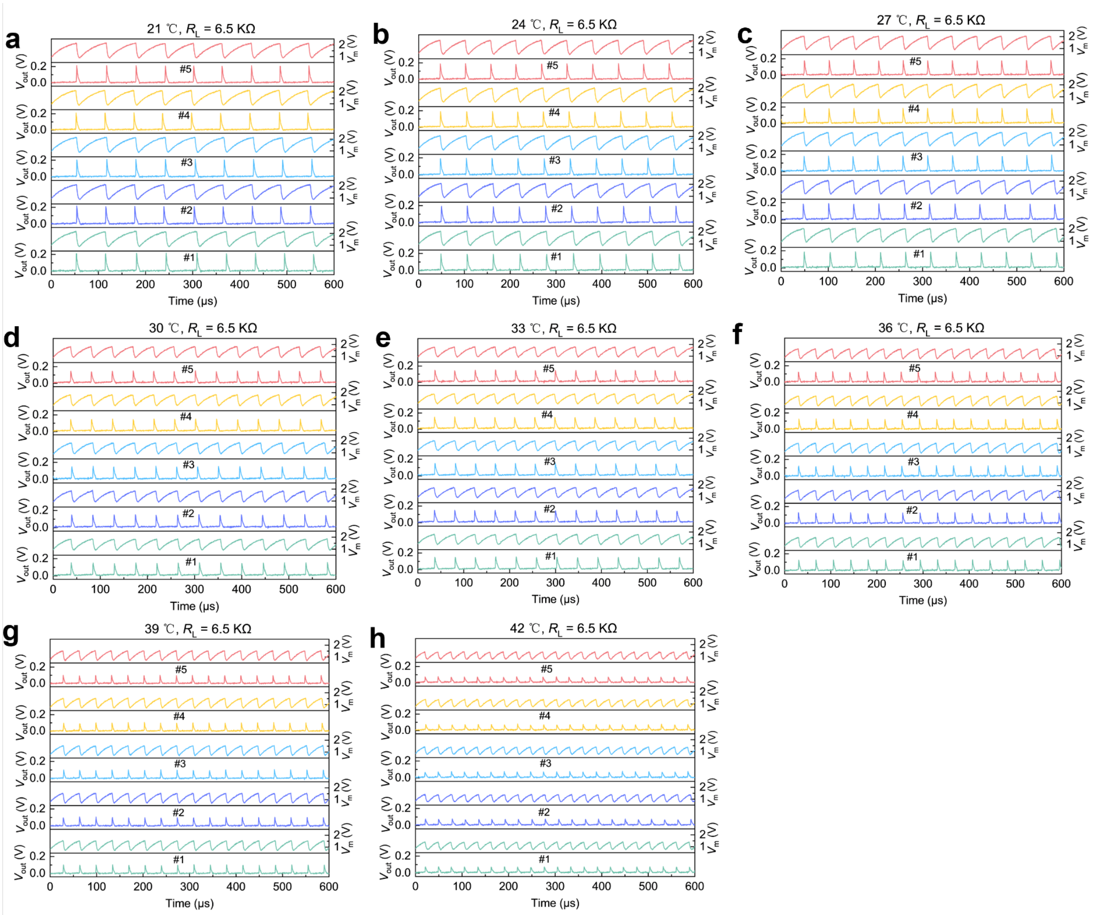

Supplementary Figure 19. The output responses of the spiking temperature sensory neuron under different temperatures with series resistance R_L= 6.5 kΩ. **(a-h)** Shown here are the responses of V_m and V_{out} to different temperature (21-42 °C). Test five times under each condition.

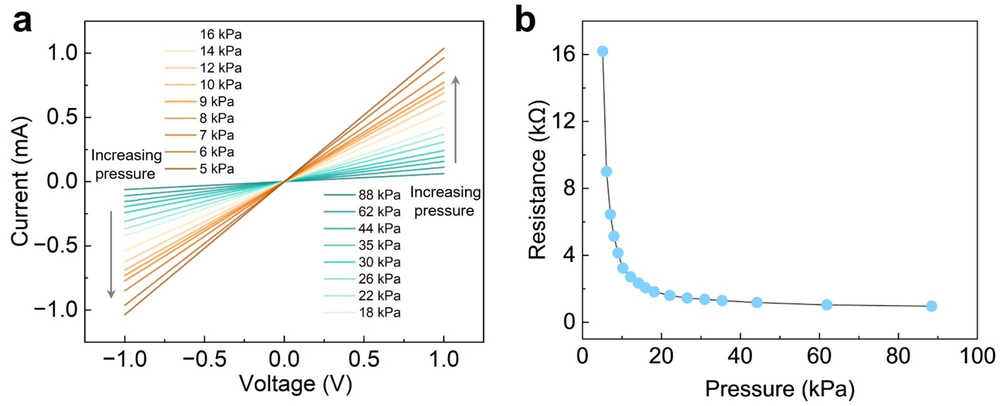

Supplementary Figure 20. Electrical characterization of the pressure sensor. (a) The typical *I*–*V* curve of the pressure sensor under various pressures, showing different electrical states in response to pressure inputs. (**b)** The relationship between the applied pressure and the resistance of the pressure sensor. As external pressure is applied, the resistance of the pressure sensor decreases exponentially. Application of pressure from ~5 to ~88 kPa results in a change in the sensor resistance from ~1 to ~16 kΩ, revealing a wide range of resistance output.

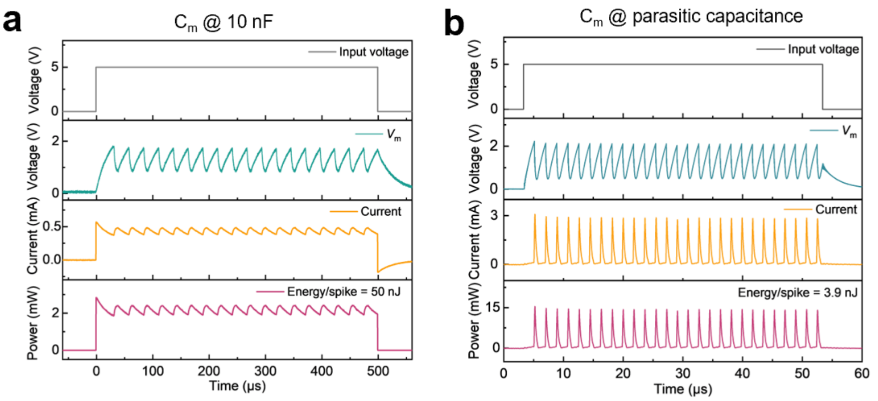

Supplementary Figure 21. The energy consumption of the CSSN for each spike event. Shown here are the curves of output voltage, output current, and power to an input voltage pulse for neuronal circuits with different capacitance: **(a)** where C_m is 10 nF, and **(b)** where C_m is the parasitic capacitance. The transient power is calculated by multiplication of input voltage with output current, and the energy consumption for each spike is calculated by dividing the total energy consumption by the spike number.

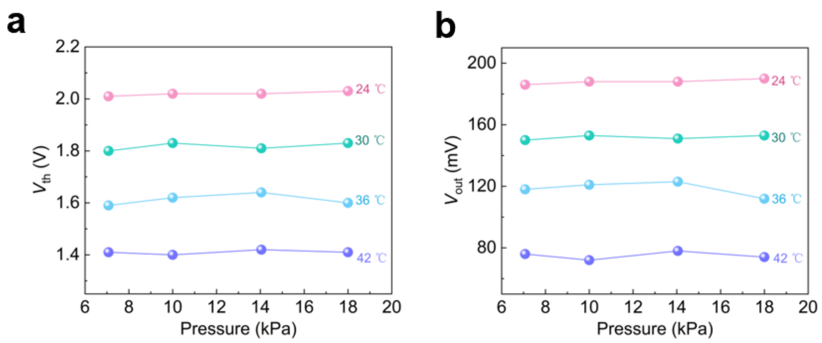

Supplementary Figure 22. Variation curves of the V_{th} (a) and the output spike amplitude (V_{out}) (b) at different pressures and temperatures.

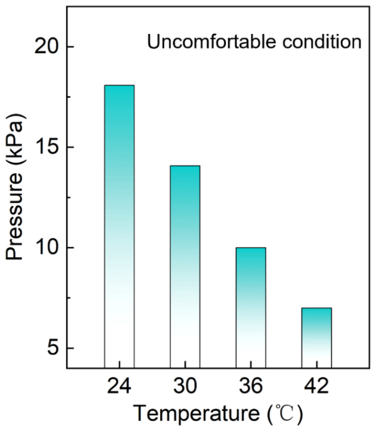

Supplementary Figure 23. Relationship between the threshold of pressure and temperature for uncomfortable condition.

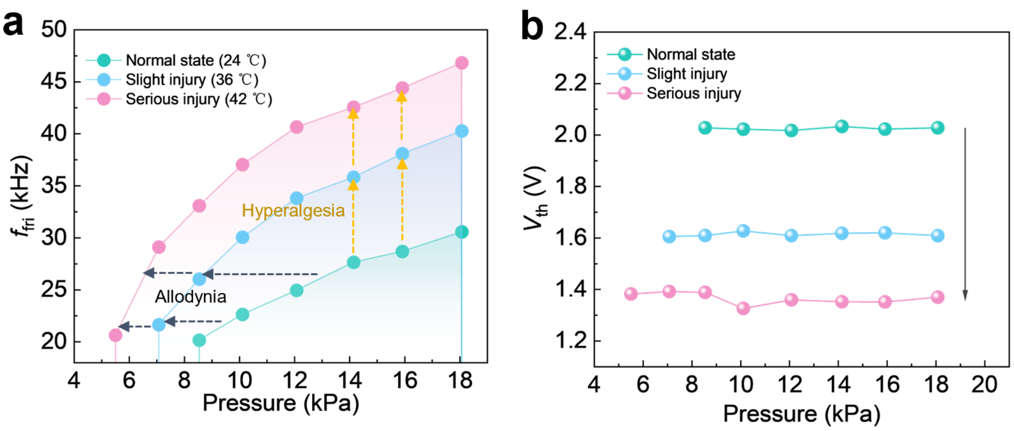

Supplementary Figure 24. Basic demonstration of the multimodal nociceptor neuron. (a) The sensitization in the artificial nociceptors characterized by hyperalgesia and allodynia. **(b)** Threshold voltage of the device at different injury state. Additional discussions are provided in Supplementary Note 3.

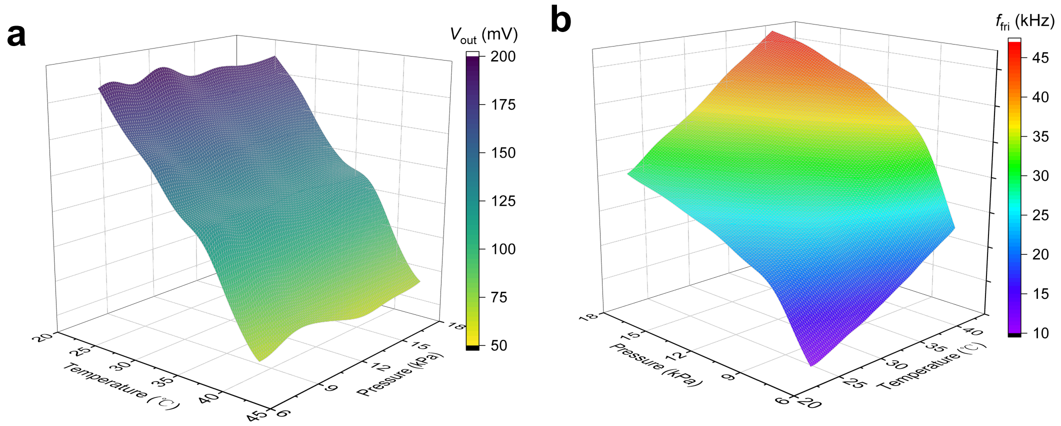

Supplementary Figure 25. The CSSN model, derived from experimental data, uses interpolation to establish a relationship between the spike amplitude (a)/frequency (b) and the perceived temperature and pressure.

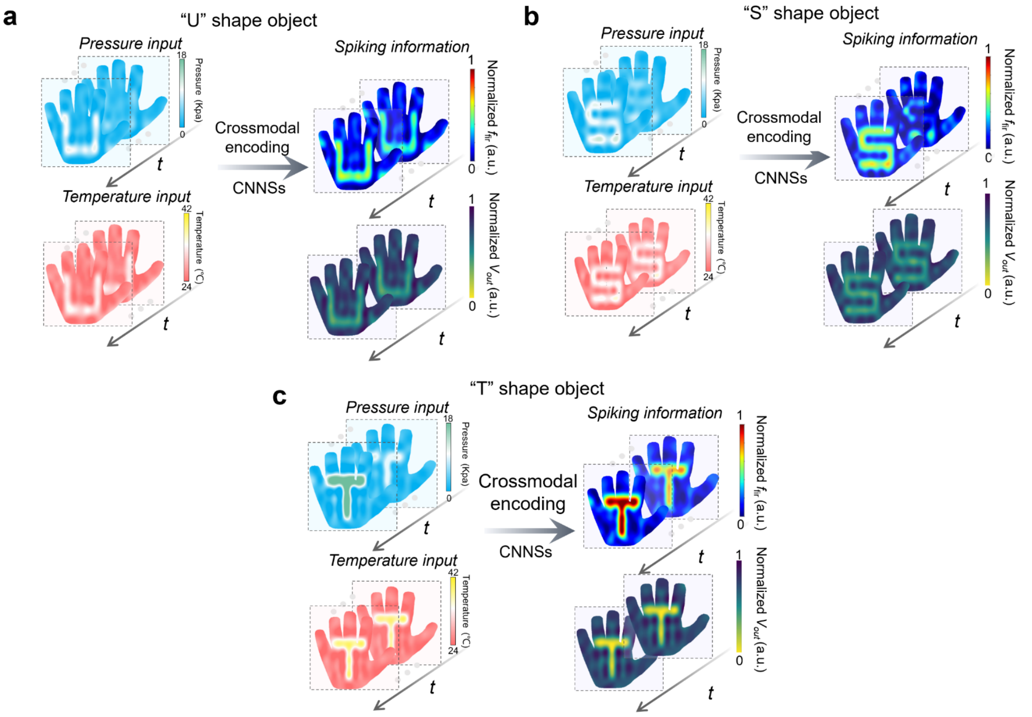

Supplementary Figure 26. (a-c) Dynamic encoding results of three objects (U, S, and T-shape) with different temperatures and pressures by the CSNNs. To demonstrate the dynamic pressing process, the pressure on each object is increased over time. The temperature of each object is changed over time in the range of 21 to 42 °C.

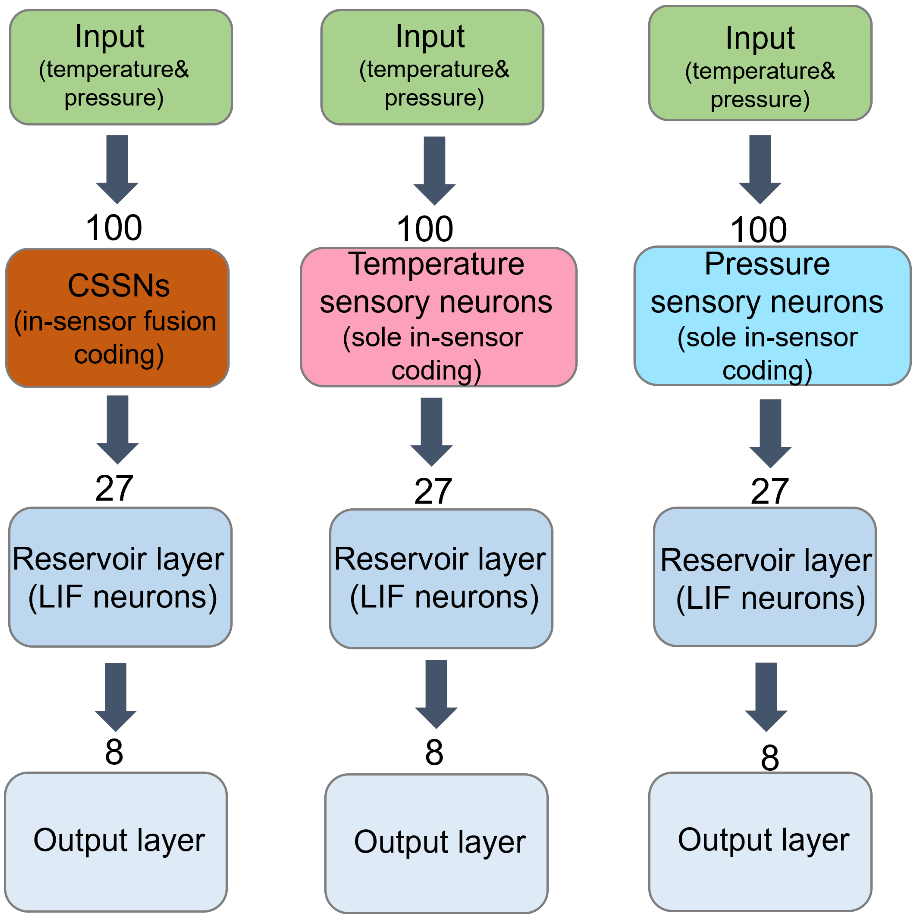

Supplementary Figure 27. Architectures of three recognition strategies: pressure-temperature in-sensor fusion coding (left), single-mode temperature in-sensor coding (middle), and single-mode pressure in-sensor coding (right).

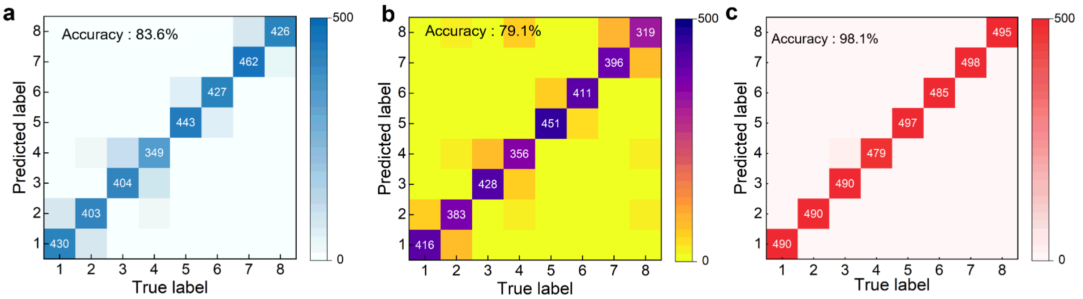

Supplementary Figure 28. Confusion matrices between three recognition strategies: sole pressure (a), sole temperature (b), and pressure-temperature confusion (c).

Supplementary Tables

2-Theta	d(Å)	I(f)	(h k l)
PDF#43-1051: VO_2 Monoclinic P21/c			
27.795	3.2070	100.0	(0 1 1)
37.088	2.4220	30.0	(2 0 0)
39.714	2.2677	10.0	(0 2 0)
44.645	2.0280	6.0	(0 2 1)
57.424	1.6034	16.0	(0 2 2)
PDF#38-1479: Cr_2O_3 Hexagonal, R-3c			
33.596	2.6653	100.0	(1 0 4)

Supplementary Table 1. X-ray diffraction (XRD) peaks of VO_2 films grown on a Cr_2O_3 buffer layer compared to standard PDF cards.

**Device**	**Flexibility**	**Forming-free**	**Distribution of V_{th}**	**C_v (V_{th})**	**Number of device (yield)**	**Ref.**
Ta/NbO_x/TiN	No	No	-1.57±0.17 V	6.51%	20	6
Au/VO_2/Au	No	Yes	1.23±0.12 V	5.32%	10	7
Pt/NbO_x/Pt	Yes	No	1.8±0.35 V	/	5	8
Ag/OIHP/ITO	Yes	/	0.3±0.1 V	/	20	9
Ag/FLBP-$CsPbBr_3$/ITO	Yes	/	1.15±0.46 V	19.74%	400 (84.3%)	10
Ag/protein nanowire/Pt	Yes	No	65±14 mV	/	117	11
Au/Cr_2O_3/VO_2/Au	Yes	Yes	1.84±0.11 V	3.73%	225 (97.8%)	**This work**

Supplementary Table 2. Comparison of D2D variations in our flexible memristor with those reported in existing works[6-11]. This analysis encompasses various parameters, including D2D variations, highlighting crucial metrics such as the threshold voltage (V_{th}) distribution and its coefficient of variation (C_v), as well as overall device yield. This comprehensive comparison clearly demonstrates the superior performance of our flexible devices in terms of consistency and reliability.

Neuron device	Flexibility	Crossmodal sensory	Sensory signal	Highest spike frequency	Energy/ spike	Ref.
Au/VO_2/Au memristor	No	No	Pressure, optical, curvature, temperature (near-sensor)	1.3 MHz	2.9 nJ	7
Ag/FLBP-$CsPbBr_3$/ITO memristor	Yes	No	Optical (in-sensor)	25 Hz	2.1-20.3 nJ	10
Ag/protein nanowire/Pt memristor	Yes	Yes	Optical, pressure, humidity (near-sensor)	0.25 Hz	~14 nJ*	11
Si/NbO_x/TiN memristor	No	No	Pressure (near-sensor)	1.1 MHz	38 pJ	12
Pt/V_3O_5/Pt memristor	No	No	Optical (in-sensor)	82 kHz	~190 nJ*	13
Ag/TaO_x/ITO memristor	No	No	Optical (near-sensor)	200 Hz	~2.5 nJ*	14
Ag/TaO_X/AlO_X /ITO memristor	No	No	Temperature (in-sensor)	1.6 kHz	~0.15 nJ*	15
Organic electrochemical transistor	No	No	Physiological signals (in-sensor)	55 Hz	~0.5 μJ*	16
Au/Cr_2O_3/VO_2/ Au memristor	Yes	Yes	Pressure, temperature (in-sensor)	650 kHz	3.9-50 nJ	**This work**

*The energy consumption per spike is calculated approximately from the P-t, V–t and I–t curves in these reference papers, respectively.

Supplementary Table 3. Comparisons with other state-of-the-art memristor-based sensory spiking neuron[7, 10-16]. To demonstrate the high-efficiency coding capabilities of the CSSN as a sensory neuron, we compared several key characteristics of artificial sensory neurons. These characteristics include the type of neuron device, crossmodal sensory, sensory modality, highest spike frequency, and energy per spike.

Ref.	This work	Nature 2019, 575, 473-479. [50]	Sci. Robot. 2019, 4, eaax2198. [52]	Nat. Commun. 2020, 11, 4602. [53]	Nat. Electron. 2021, 4, 429-438. [51]	Sci. Robot. 2022, 7, eabl7286. [36]	Nat. Commun. 2022, 13, 79. [4]	Science 2023, 380, 735-742. [54]
**Sensory modality**	Pressure & temperature	Pressure	Pressure & temperature	Pressure & temperature	Pressure	Pressure	Pressure & olfactory	Pressure & temperature
**Sensor units**	Pressure sensor + VO_2 memristor	Pressure sensor	Pressure sensor + temperature sensor	Pressure sensor+ photodetector	Tactile sensor	Pressure sensor	Pressure sensor + olfactory sensor	Pressure sensor + temperature sensor
**Encoding circuits**	Compact VO_2 neuronal circuit	Commercial ADC chip	Commercial ADC chip	Compact synaptic transistor circuit	Commercial ADC chip	CMOS neuronal circuit + synaptic transistor	Commercial ADC chip	Ring oscillator + edge detector
**Sensory informatio n encoding to spike-trains**	Yes	No	Yes	No	Yes	Yes	No	Yes
**In-sensor encoding**	Yes	No	No	No	No	No	No	No
**Function**	Human–machine interaction	Human–machine interaction	Human–machine interaction	Human–machine interaction	Biological sensorimotor loop	Human–machine interaction	Human–machine interaction	Biological sensorimotor loop

Supplementary Table 4. Comparison of our flexible haptic-feedback system with existing works.

Supplementary Notes

Supplementary Note 1: Characteristics of the VO_2 film deposited on Cr_2O_3 buffer film at a low temperature.

The Cr_2O_3 was chosen as the structural buffer layer to reduce the deposition temperature of VO_2 film to 280 °C, compatible with flexible circuits. This is because Cr_2O_3 (hexagonal, a = 0.496 nm, c = 1.359 nm) shows small lattice mismatch with the rutile (R) VO_2 (tetragonal, a = 0.455 nm, c = 0.286 nm)[17,18]. Meanwhile, Cr_2O_3 thin films can be readily deposited at low temperature (≥30 °C) due to the low formation enthalpy of the material ($\Delta H_{Cr_2O_3}$ = −1129 kJ/mol)[19]. We deposited Cr_2O_3 film with excellent crystallinity at 280 °C (Supplementary Fig. 2a). The VO_2 (R) with high crystallization quality is then grown on the Cr_2O_3 film. This VO_2 (R) thin film changes to VO_2 (M) when the temperature is cooled down to room temperature. The microstructure of the grown VO_2 films was systematically investigated. According to the XRD spectrum, the as-grown VO_2 film with buffer layer is polycrystalline, including the (011), (200), (020), (021), and (022) planes of VO_2 (M), which has a structure with the $P2_1/c$ space group (JCPDS card 43-1051), and the peak of (011) planes is significantly improved with the increase of buffer layer thickness, as shown in Supplementary Fig. 2b and Supplementary Table 1. Supplementary Fig. 2c shows the TEM image of the VO_2 films. The crystallinity of the VO_2 films deposited on a 40 nm-Cr_2O_3 buffer layer is significantly improved compared to the one without buffer layer. These results indicate the Cr_2O_3 buffer layer promotes the crystallization of VO_2 (M) at low growth temperature. The low average root mean square (RMS) roughness (~3 nm) of five randomly selected areas on the film reveals that the VO_2 films are smooth with fine crystallization (Supplementary Fig. 4). No diffusion of Cr element into VO_2 film is found according to the cross-section element mapping by EDS shown in Supplementary Fig. 5. To investigate the metal-to-insulator transition (MIT) behavior of VO_2 films, we studied the temperature-dependent resistance of the thin film (Supplementary Fig. 6). It is found that the VO_2 film deposited on Cr_2O_3 shows a much stable transition from M phase to R phase compared to the one without buffer layer. This stable MIT transition

is also been verified by the temperature-dependent Raman experiment where the detailed results are shown in Supplementary Fig. 7.

Supplementary Note 2: Spiking behaviors of the VO_2 neurons.

The threshold switching (TS) characteristics of our memristors enable the neural firing when adopting suitable circuit parameters (input voltage (V_{in}), load resistor (R_L), and capacitance (C_m)) (Supplementary Fig. 16a). As shown in Supplementary Fig. 18b, the process of charging and discharging can be observed clearly. The peak and trough values of the neuronal oscillation curves correspond to V_{th} and V_{hold}, respectively. The spiking frequency (f_{fir}) of the artificial neuron can be configured by different V_{in} and R_L (see Supplementary Fig. 16c, d). As the R_L increases, the input current gradually decreases and the charge accumulation becomes slow, the f_{fir} gradually decreases. In contrast, as the applied V_{in} increases, the f_{fir} gradually rises. The f_{fir} range can be further extended by modifying parallel (C_m) (Supplementary Fig. 16e). As the parallel capacitance increases, larger C_m makes the integration process slower. the f_{fir} gradually decreases. The artificial neuron still exhibits stable neuronal oscillation properties after 6 hours with a spiking period of < 30 μs (Supplementary Fig. 16f). These rich strength-modulated spiking behaviors are essential for the construction of artificial spiking sensing systems.

Supplementary Note 3: "Sensitization" of the artificial nociceptor under injury.

In biological nociceptor, sensitization is a physiological phenomenon that enhances the pain sensitivity of sensory neurons to noxious stimuli[20,21]. This often manifests in two primary forms: allodynia, where a normally innocuous stimulus becomes painful, and hyperalgesia, where the response to a noxious stimulus becomes more pronounced. Allodynia is characterized by a decreased response threshold to the stimulus, while hyperalgesia is marked by an increased response intensity to the same stimulus. For example, when skin is wounded and becomes bruised, it becomes highly responsive to subsequent stimuli, even a gentle touch, as a protective action to prevent further harm

to the injured tissue.

To demonstrate the "sensitization" feature of our CSSN, we applied different temperature to the VO_2 devices, simulating "injury" to the artificial nociceptor system. As shown in Supplementary Fig. 24a, the output f_{fri} under different input pressures was recorded for devices that experienced various levels of "injury". The results show that the "injured" CSSN exhibited a higher f_{fri} for the same applied pressure, indicating the hyperalgesia characteristic. Notably, the pressure threshold shifted lower because the V_{th} of the device decreased with increasing temperature (Supplementary Fig. 24b). In comparison to the normal state, a smaller pressure threshold could activate the injured CSSN, replicating the allodynia characteristic. Interestingly, the damage to the artificial nociceptor is recoverable because the VO_2 device's damage (cooling process, Supplementary Fig. 17c) is reversible, corresponding to a transient skin allergy.

Supplementary Note 4: Robotic crossmodal sensing-feedback experiment.

To demonstrate the crossmodal sensing-feedback functions of the CSSNs, we designed a simple "open and grasp" motion as a proof of concept to perform the feedback-control of the robot. For this experiment, we developed a FPCB by integrating a CSSN circuit and a microcontroller (MCU, STM32F103C6T6) on a flexible PI substrate. The external temperature and pressure were applied to FPCB, respectively. Bimodal pressure and temperature signals were simultaneously encoded to spikes by the CSSN under the conditions of 5.0 V input pulse amplitude, 500 μs interval, and 500 μs width. When MCU receives a neural spike signal, a feedback-control command was relayed to the executing unit of the robotic arm (uHand2.0) via WiFi module. Inspired by the biological reflex system, two threshold f_m and f_{fir0} were set. When the applied stimulations above threshold f_m and f_{fir0}, the robotic arm will trigger grasp and avoidance (i.e., opening) behavior, respectively. The threshold needs to be chosen properly by adjusting circuit parameters to avoid incorrect triggering noise signals.

Supplementary Note 5: Simulation details about the crossmodal in-sensor spiking

reservoir computing system simulations.

We developed a custom dataset comprising 1,200 samples for training and testing a neural network, designed to replicate the tactile interaction of a human hand pressing objects shaped as “H”, “U”, “S”, and “T”. Each sample captures a pressing action with gradual increases in temperature and pressure, consisting of 100 distinct sensory pixels. These pixels record specific instances within the pressing sequence along with corresponding temperature and pressure data over time. To mimic real-world conditions and assess network robustness, we systematically introduced Gaussian noise of varying intensities (δ/μ = 0, 0.01, 0.02, 0.04, 0.08) across the dataset. The dataset was split equally into 600 samples for training and 600 for testing.

We implemented a crossmodal in-sensor spiking reservoir computing system in Python 3.9, comprising a sensory layer, a reservoir layer, and a readout layer based on experimental results. In the sensory layer, a 100-node CSSN array simulates the human hand, encoding dynamic pressure and temperature into spike amplitude and frequency signals. The reservoir layer consists of 27 LIF neurons. Connectivity among neurons is randomly established, ensuring a dynamic and rich interaction pattern conducive to complex information processing. Neurons receive spike signals from CSSN nodes, with random synaptic connections promoting diverse neural interactions. Neuronal connectivity follows geometric rules, with synaptic weights uniformly set at 12 for consistent signal processing. The membrane potential dynamics of LIF neurons are described by the equations:

$$u_{o}(t)=u_{o}(t\text{-}\Delta t)\exp\left(-\frac{\Delta t}{RC}\right)+U(t) \tag{1}$$

$$s_{o}(t)=\vartheta(u_{o}(t))=\begin{cases}1, & u_{o}(t)\geq V_{th}\\ 0, & u_{o}(t)<V_{th}\end{cases} \tag{2}$$

$$u_{o}(t)=\begin{cases}0, & \text{if } s_{o}(t)\\ u_{o}(t), & \text{else}\end{cases} \tag{3}$$

The first term in equation (1) represents a leaky process, where $u_{o}(t\text{-}\Delta t)$ is the membrane of output neuron o at $t\text{-}\Delta t$ step, RC is a leaky constant. $U(t)$ is event signal from input neuron and other LIF neurons in the reservoir. Eq. (2) represents fire behaviour, in which ϑ is a step function, when $u_{o}(t)\geq V_{th}$, neuron o fires and generates

a spike $s_o(t)$. V_{th} is the neuronal firing threshold voltage. Eq. (3) represents reset behaviour, if neuron o fires, the membrane resets to zero, otherwise it stays unchanged. In our experiments, the V_{th} is 0.18 V, and the RC is 20 μs.

The readout layer comprises a fully connected network of 27 neurons linked to eight output labels. These labels correspond to four different object shapes, each represented under high and low input intensities. This layer undergoes training using a backpropagation (BP) algorithm, adapted with a sigmoid-type surrogate gradient function to accommodate the non-differentiable nature of spiking neurons. The learning process optimizes classification accuracy based on a cross-entropy loss function calculated from the spiking rates and the assigned labels of the output neurons. This structured approach ensures precise recognition of multimodal sensory data, substantiating the network's capability in handling complex sensory integrations.

Supplementary References

1. Zhang, S. Kim, I. & Lauhon, L. Stoichiometry engineering of monoclinic to rutile phase transition in suspended single crystalline vanadium dioxide nanobeams. *Nano lett.* **11**, 1443-1447 (2011).

2. Miao, P. Wu, J. Du, Y. Sun, Y. & Xu, P. Phase transition induced Raman enhancement on vanadium dioxide (VO_2) nanosheets. *J. Mater. Chem. C* **6**, 10855-10860 (2018).

3. Kim, H. *et al.* Flexible thermochromic window based on hybridized VO_2/Graphene. *ACS Nano* **7**, 5769-5776 (2013).

4. Liao, F. et al. Ultrasensitive flexible temperature-mechanical dual-parameter sensor based on vanadium dioxide films. *IEEE Electron Device Lett.* **38**, 1128-1131 (2017).

5. Lou, Z., Wang, L., Jiang, K., Wei, Z. & Shen, G. Reviews of wearable healthcare systems: Materials, devices and system integration. *Mater. Sci. Eng: R* **140**, 100523 (2020).

6. Li, F. et al. A skin-inspired artificial mechanoreceptor for tactile enhancement and integration. *ACS Nano* **15**, 16422-16431 (2021).

7. Yuan, R., et al. A calibratable sensory neuron based on epitaxial VO_2 for spike-based neuromorphic multisensory system. *Nat. Commun.* **13**, 3973 (2022).

8. Ang, J. M., Dananjaya, P. A., Ang, C. C. I., Lim, G. J. & Lew, W. S. Strain-induced degradation and recovery of flexible NbO_x-based threshold switching device. *Sci. Rep.* **13**, 16000 (2023).

9. Tang, L. et al. Flexible threshold switching selectors with ultrahigh endurance based on halide perovskites. *Adv. Electron. Mater.* **8**, 2100771 (2021).

10. Wang, Y. et al. Memristor-based biomimetic compound eye for real-time collision detection. *Nat. Commun.* **12**, 5979 (2021).

11. Fu, T. et al. Self-sustained green neuromorphic interfaces. *Nat. Commun.* **12**, 3351 (2021).

12. Zhang, X. et al. An artificial spiking afferent nerve based on mott memristors for neurorobotics. *Nat. Commun.* **11**, 51 (2020).

13. Nath, S. K. et al. Optically tunable electrical oscillations in oxide-based memristors for neuromorphic computing. *Adv. Mater.* 2400904 (2024).

14. Chen, C. et al. A photoelectric spiking neuron for visual depth perception. *Adv. Mater.* **34**, 2201895 (2022).

15. Shi, K. et al. An oxide based spiking thermoreceptor for low-power thermography edge detection. *IEEE Electron Device Lett.* **43**, 2196-2199 (2022).

16. Sarkar, T. et al. An organic artificial spiking neuron for in situ neuromorphic sensing and biointerfacing. *Nat. Electron.* **5**, 774-783 (2022).

18. Dillemans, L. Smets, T. Lieten, R. Menghini, M. Su, C. & Locquet, J. Evidence of the metal-insulator transition in ultrathin unstrained V_2O_3 thin films. *Appl. Phys. Lett.* **104**, 071902 (2014).

19. Jin, P. Nakao, S. Wang, S. & Wang, L. Localized epitaxial growth of α-Al_2O_3 thin films on Cr_2O_3 template by sputter deposition at low substrate temperature. *Appl. Phys. Lett.* **82**, 1024-1026 (2003).

20. Gold, M. S. & Gebhart, G. F. Nociceptor sensitization in pain pathogenesis. *Nat. Med.* **16**, 1248-1257 (2010).

21. Jensen, T. S. & Finnerup, N. B. Allodynia and hyperalgesia in neuropathic pain: Clinical manifestations and mechanisms. *Lancet Neurol.* **13**, 924-935 (2014).
