# Supplementary material for: Crossmodal sensory neurons based on high-performance flexible memristors for human-machine in-sensor computing system
Source: Nat Commun. 2024 Aug 23;15:7275. doi: 10.1038/s41467-024-51609-x (PMC11344147; doi:10.1038/s41467-024-51609-x)
Supplement: Supplementary file 3 — Description Of Additional Supplementary File [file 41467_2024_51609_MOESM3_ESM.pdf]

Description of Additional Supplementary File

Supplementary Movie 1. The flexible integrated spiking sensing-feedback system performing haptic-feedback for human-machine interaction.
